# A heat-killed preparation of mycobacterium obuense can reduce metastatic burden *in vivo*

**DOI:** 10.1186/2051-1426-2-S3-P54

**Published:** 2014-11-06

**Authors:** Daniel Fowler, Angus Dalgleish, Wai Liu

**Affiliations:** 1 St George's University of London, United Kingdom

## Introduction

Immune deficiency has recently been identified as one of the hallmarks of cancer, and as such, strategies that rectify this are therapeutically attractive. A potential immunotherapy is IMM-101, which is a suspension of heat-killed *Mycobacterium obuense *that has recently undergone a Phase I trial of safety and tolerability in melanoma patients (NCT01559819). It is also currently undergoing Phase II trials in pancreatic cancer in combination with Gemcitabine (NCT01303172) and colorectal cancer in combination with radiation therapy (NCT01539824).

## Methods

In the current study, two *in vivo *models were employed to discern the action of IMM-101 on tumour growth and metastatic potential. In the first model, either a colorectal (CT26) or a melanoma (B16F10) tumour, sygeneic to the BALB/c and C57BL/6 mouse, were used to assess the effect of IMM-101 administered subcutaneously on tumour growth. In the second model, CT26 were inoculated into the tail vein of a BALB/c mouse to model the effects of the agent on the metastatic potential of the tumour. Methodologically, BALB/c mice were primed for three weeks with CT26 lysate intraperitoneally and IMM-101 subcutaneously prior to an intravenous challenge with viable CT26 cells. The mice were then administered with therapeutic subcutaneous IMM-101 for the following three weeks, and metastatic burden in the lungs measured on day 42.

## Results

Results from the subcutaneous model showed IMM-101 had no significant effect on tumour growth in both the colorectal and melanoma tumours. For example, mean +/- SD of the area under curves for the growth of CT26 tumours in control and IMM-101 treatment groups were 13,798 +/- 4,608 and 20,877 +/- 10,259, respectively; p = 0.062. However, in the second model, there was a significant reduction in the number of lung metastatic lesions in those mice primed with tumour lysate and IMM-101 and then further treated with IMM-101 (mean +/- SD of the number of lung metastases were 1.8 +/- 2.9 vs. 19 +/- 18 for controls where mice were only primed with tumour lysate; p < 0.01). Furthermore, splenocytes harvested from these mice displayed significantly enhanced production of IFN-g, IL-17 and GMCSF in response to in vitro stimulation with anti-CD3.

## Conclusions

These data from *in vivo *models suggest that IMM-101 has a greater effect on the metastatic capacity of tumour cells rather than on overall tumour growth. Intriguingly, this mimics other pre-clinical observations that suggest IMM-101 is efficacious against metastasis.

